# The effect of type 2 diabetes on the prognosis of community-acquired pneumonia

**DOI:** 10.3389/fendo.2026.1878364

**Published:** 2026-07-14

**Authors:** Fan-Shuo Kong, Long-Ye Zhang, Rui Wang, Chun-Ming Ma

**Affiliations:** 1 Department of Endocrinology, The First Hospital of Qinhuangdao, Qinhuangdao, Hebei, China; 2 Department of Nephrology, The First Hospital of Qinhuangdao, Qinhuangdao, Hebei, China

**Keywords:** community-acquired pneumonia, in-hospital mortality, respiratory failure, septic shock, type 2 diabetes mellitus

## Abstract

**Objective:**

The aim of the study was to explore the effects of type 2 diabetes mellitus (T2DM) on the severity and prognosis in community-acquired pneumonia (CAP) patients.

**Methods:**

A retrospective analysis was carried out on 2471 CAP patients. All subjects were divided into the non-diabetic group and the T2DM group. The information of pneumonia and mortality records were collected.

**Results:**

This study enrolled 2471 CAP patients. Of these patients, 559 (22.6%) had T2DM. The frequencies of septic shock (6.4% vs 3.5%, *χ*^2^ = 9.759, *P* = 0.002) and respiratory failure (22.7% vs 16.5%, *χ*^2^ = 11.482, *P* = 0.001) were higher in patients with T2DM than patients without T2DM. The mortality was higher in patients with T2DM than patients without T2DM (T2DM 8.4% vs non-diabetes 4.3%, *χ*^2^ = 14.353, *P* < 0.001). In multiple logistic regression analysis, T2DM was an independent risk factor of death in CAP inpatients (OR = 1.713, 95%CI:1.157~2.536, *P* = 0.007). Stratified by CURB-65 risk grade, T2DM was an independent risk factor for in-hospital mortality in patients with low risk group (OR = 2.150, 95%CI: 1.092–4.233, *P* = 0.027) and intermediate risk group (OR = 1.966, 95%CI: 1.066–3.626, *P* = 0.030), yet no significant difference was seen in the high-risk group (OR = 1.133, 95%CI: 0.563–2.280, *P* = 0.727).

**Conclusion:**

CAP patients with T2DM were more severe than CAP patients without T2DM. The mortality was higher in CAP patients with T2DM compared with non-diabetic patients, especially for patients with low to intermediate risk.

## Introduction

Community-acquired pneumonia (CAP) is defined as pneumonia that is acquired outside the hospital. Epidemiological surveys in the United States show that the incidence of adult CAP is 24.8 per 10,000 people per year (1). In France, the incidence of adult CAP is 4.7 per 1,000 people per year (2). The incidence of adult CAP in three cities in South America ranged from 1.76 to 7.03 per 1,000 people per year (3). Epidemiological surveys in South Korea indicated that the incidence of adult CAP was 307.7 per 100,000 people per year (4). It is evident that CAP is a significant global health issue and the most common type of infection among patients with lower respiratory tract infections.

CAP not only has a high incidence rate but also increases the likelihood of hospital admission, imposing a heavy economic burden on both patients and governments. Compared with outpatients, hospitalized CAP patients incur higher medical costs (5). A survey in New York revealed that a total of 4,614,108 hospitalizations occurred between 2010 and 2014, with pneumonia accounting for 6.2% of these cases. More than half of these pneumonia patients had CAP (6). A retrospective cohort study in the United States showed that hospitalized CAP patients had an average length of stay of 5.7 days and an average hospitalization cost of $17,736. Among these patients, 22.7% were transferred to the intensive care unit (ICU) for treatment. The 30-day and 180-day readmission rates were 8.8% and 20.1%, respectively (7). Data from the United States Veterans Health Administration showed that annual CAP-related medical expenditures reached $750 million (8). Results from South Korean Health Insurance and Review Assessment database indicated that between 2009 and 2013, the annual incidence of CAP-related hospitalization was 626 per 100,000 people. Over these five years, the average direct medical cost per person due to CAP hospitalization was $1,851 (9).

Meanwhile, CAP is the sixth leading cause of death worldwide and one of the major diseases threatening patients’ lives (10). Due to differences in study populations, regions, medical environments, and other factors, the mortality rates of CAP patients reported in various studies vary significantly. Overall, the mortality rate of CAP patients exhibits the following characteristics: Firstly, higher mortality rates were observed in elderly patients, especially those of advanced age (11, 12); Secondly, hospitalized CAP patients showed higher mortality rates (13); Thirdly, the mortality rates increased in CAP patients with underlying diseases (14).

Diabetes mellitus is another major disease currently threatening global health. According to the latest epidemiological data from the International Diabetes Federation (IDF) in 2019, the global number of adults with diabetes has reached 463 million, with type 2 diabetes mellitus (T2DM) being the most common form (15). As early as the 1990s, some scholars proposed that the lung is an additional target organ for diabetic damage, and pneumonia can be considered a pulmonary complication caused by diabetes (16, 17). Epidemiological surveys show that diabetic patients have an increased risk of CAP. A systematic review indicated that diabetic patients have a 40% higher risk of CAP, and their risk of pneumococcal pneumonia is 2.3 times that of non-diabetic patients (18). Key drivers include hyperglycemia-induced immune paralysis, pulmonary microangiopathy, ferroptosis, glycation and methylation changes, and gut-lung dysbiosis. Host-targeted therapy with moderate glycemic control, continued metformin, and pathogen-directed antibiotics improves survival (19). However, research findings on the prognosis of T2DM patients with concurrent CAP are inconsistent (20, 21).

This study used medical record data of hospitalized CAP patients from the First Hospital of Qinhuangdao between 2015 and 2018 to explore the impact of T2DM on the hospitalization costs, disease severity, and in-hospital prognosis of CAP patients.

## Methods

We conducted a retrospective study. All the subjects were patients from the First Hospital of Qinhuangdao between January 2015 and December 2018. The inclusion criteria were as follows: all patients were hospitalized due to CAP and were aged 18 years or above. CAP was defined as pneumonia that occurred in the community setting, presents with clinical manifestations related to pneumonia, and shows new patchy infiltrates, lobar or segmental consolidation, ground-glass opacities, or interstitial changes on chest computed tomography (22). The exclusion criteria were as follows: 1.patients with type 1 diabetes or special types of diabetes mellitus; 2. patients with diabetes mellitus of unclear classification; 3. patients with prediabetes; 4. pregnant patients; 5. patients with puerperal infection; 6. clinical data about CURB-65 missing. Patients with the missing data were excluded. This research was granted approval by the Ethics Committee of the First Hospital of Qinhuangdao (ethical approval number: 2021A006). Data were retrieved from the Hospital Information System and kept confidential.

### Data collection

Relevant patient data were extracted from the hospital’s electronic medical record system. General information included gender, age, ethnicity, length of hospital stay, and hospitalization costs of the patients. Patient information related to the disease was extracted, including the following aspects: Pneumonia-related information: pneumonia diagnosis, disease onset setting, presence of respiratory failure[Partial pressure of arterial oxygen (PaO_2_) <60 mmHg, with or without partial pressure of arterial carbon dioxide (PaCO_2_) >50 mmHg], presence of septic shock[Systolic blood pressure <90 mmHg, pulse pressure <30 mmHg, accompanied by manifestations of tissue hypoperfusion], and use of mechanical ventilation. Diabetes-related information: diagnosis and classification of diabetes mellitus. Presence of underlying lung diseases: lung cancer, chronic obstructive pulmonary disease (COPD), and bronchial asthma. Diagnosis of other diseases: chronic kidney disease, hypoproteinemia, heart failure, coronary atherosclerotic heart disease, and stroke and transient ischemic attack (TIA). ICU admission status and in-hospital outcomes.

Clinical data about CURB-65 were also extracted. The scores of CURB-65 [confusion, urea>7mmol/L, respiratory rate≥30/min, blood pressure (systolic blood pressure<90mmHg or diastolic blood pressure ≤ 60mmHg) and age ≥65years] were calculated. Patients with CURB-65 score 0~1 were defined as low risk group, score 2 were defined as intermediate risk group, scores 3~5 were defined as high risk group (23).

### Grouping

Patients were divided into two groups based on the presence of T2DM: the T2DM group and the control group.

### Statistical analyses

The statistical software SPSS 24.0 for Windows was used to perform all analyses (SPSS, Inc., Chicago, IL). Measurement data were expressed as mean ± standard deviation. Categorical data were expressed as case numbers and percentages. The t-test was used for comparing measurement data between groups, and the chi-square (*χ*^2^) test was used for comparing categorical data. For non-normally distributed data, median and interquartile range were used, and Mann-Whitney U test was used for comparing measurement data between groups. The in-hospital mortality rates of CAP patients were compared between the T2DM group and the control group. Multivariate Logistic regression analysis was applied to explore the relationship between T2DM and in-hospital mortality of CAP patients. A *P*-value < 0.05 was considered statistically significant.

Propensity score matching (PSM) was also used to minimize the effect of confounding factors. A one-to-one nearest neighbor matching algorithm was applied using a caliper width of 0.05. The following variables were selected to generate the propensity score: age, gender, ethnicity, and CURB-65. Finally, 559 matched pairs were generated and applied to further analyses.

## Results

A total of 2,682 hospitalized cases of CAP were included in this study. Among them, 36 cases were excluded for having type 1 diabetes mellitus, special types of diabetes mellitus, or diabetes mellitus with unclear classification; 23 cases were excluded for being in the prediabetes stage; 8 cases were excluded for being pregnant or having puerperal infection; and 144 cases were excluded for CURB-65 missing. Finally, 2,471 hospitalized CAP patients were included in the study, consisting of 1,429 males and 1,042 females, with a mean age of 66.3 ± 17.4 years.

Among the enrolled patients, 559 had T2DM, accounting for 22.6% of the hospitalized CAP patients. There were no statistically significant differences in gender and ethnicity distribution between the two groups (*P* > 0.05). The age and CURB-65 of patients in the T2DM group were higher than that in the control group (*P* < 0.001). No statistically significant differences were observed in the proportions of lung cancer, COPD, and asthma between the two groups (*P* > 0.05). The proportions of coronary atherosclerotic heart disease, heart failure, stroke and TIA, chronic kidney disease, and hypoproteinemia in the T2DM group were all higher than those in the control group (*P* < 0.05). The total hospitalization cost and length of hospital stay in the T2DM group were higher than those in the control group (*P* < 0.001). The rates of mechanical ventilation support and ICU admission in the T2DM group were also higher than those in the control group (*P* < 0.05) (**Table 1**).

**Table 1:** The characteristics of community acquired pneumonia inpatients with and without type 2 diabetes.

Variables		Non-diabetes group (N = 1912)	Type 2 diabetes group (N = 559)	*t* or *χ*^2^	*P*
Sex (males/females)		1109/803	320/239	0.102	0.750
Age (years)		64.7 ± 18.5	71.7 ± 11.8	10.657	<0.001
Ethnicity [n(%)]	Han	1788 (93.5)	536 (95.9)	4.368	0.113
	Other	84 (4.4)	16 (2.9)		
	Unknown	40 (2.1)	7 (1.2)		
CURB-65 Score		1.0 (0.0~2.0)	1.0 (1.0~2.0)	7.055	<0.001
CURB-65 Grade [n(%)]	Low risk group	1316 (68.8)	313 (56.0)	31.735	<0.001
	Intermediate risk group	417 (21.8)	173 (30.9)		
	High risk group	179 (9.4)	73 (13.1)		
Lung cancer [n(%)]		110 (5.8)	37 (6.6)	0.580	0.446
COPD [n(%)]		148 (7.7)	33 (5.9)	2.151	0.143
Asthma [n(%)]		40 (2.1)	16 (2.9)	1.158	0.282
CHD [n(%)]		421 (22.0)	239 (42.8)	95.009	<0.001
Heart failure [n(%)]		323 (16.9)	150 (26.8)	27.613	<0.001
Stroke and TIA [n(%)]		416 (21.8)	222 (39.7)	72.817	<0.001
CKD [n(%)]		42 (2.2)	67 (12.0)	98.298	<0.001
Hypoproteinemia [n(%)]		362 (18.9)	129 (23.1)	4.665	0.031
Hospital length of stay [days]		9.0 (7.0~13.0)	11.0 (8.0~16.0)	7.955	<0.001
Hospital cost [CNY]		10848 (7182~19103)	16170 (10423~28679)	10.413	<0.001
Mechanical ventilation [n(%)]		186 (9.7)	73 (13.1)	5.115	0.024
ICU admissions [n(%)]		236 (12.3)	97 (17.4)	9.308	0.002
Septic shock [n(%)]		66 (3.5)	36 (6.4)	9.759	0.002
Respiratory failure [n(%)]		315 (16.5)	127 (22.7)	11.482	0.001
Death [n(%)]		83 (4.3)	47 (8.4)	14.353	<0.001

Numerical data were expressed as mean ± SD, and when not normally distributed, they were expressed as medians (IQR) and Mann-Whitney U test was used for comparing measurement data between groups. COPD, chronic obstructive pulmonary disease; CHD, coronary heart disease; TIA, transient ischemic attack; CKD, chronic kidney disease; CNY, China Yuan; ICU, intensive care unit; SD, standard deviation; IQR, interquartile range.

A total of 102 patients developed septic shock in this study, accounting for 4.1% of the study population. The incidence of septic shock was 3.5% in non-diabetic CAP patients and 6.4% in T2DM patients. The incidence of septic shock in T2DM patients was significantly higher than that in non-diabetic patients (*χ*^2^ = 9.759, *P* = 0.002). Multivariate logistic regression analysis showed that T2DM was an independent risk factor for septic shock in hospitalized CAP patients (OR = 1.648, 95% CI: 1.057–2.569, *P* = 0.028) (**Table 2**). CURB-65 and hypoproteinemia were also independent risk factors for septic shock in hospitalized CAP patients.

**Table 2:** The risk factors of septic shock in community acquired pneumonia inpatients.

Variable		OR	95%CI	*P*
CURB-65	Low risk group	1		
	Intermediate risk group	2.647	1.487~4.711	0.001
	High risk group	14.040	8.305~23.736	<0.001
Hypoproteinemia		2.605	1.696~4.002	<0.001
Type 2 diabetes		1.648	1.057~2.569	0.028

Multiple logistic regression analysis, septic shock was considered as the dependent variables in a multiple logistic regression analysis with sex, age, ethnicity, CURB-65, type 2 diabetes, lung cancer, COPD, asthma, CHD, heart failure, CKD, hypoproteinemia, stroke and TIA as independent variables. OR, odds ratio; CI, confidence interval; COPD, chronic obstructive pulmonary disease; CHD, coronary heart disease; CKD, chronic kidney disease; TIA, transient ischemic attack.

A total of 442 patients developed respiratory failure in this study, accounting for 17.9% of the study population. The incidence of respiratory failure was 16.5% in non-diabetic CAP patients and 22.7% in T2DM patients. The incidence of respiratory failure in T2DM patients was significantly higher than that in non-diabetic patients (*χ*^2^ = 11.482, *P* = 0.001). Multivariate logistic regression analysis indicated that T2DM was an independent risk factor for respiratory failure in hospitalized CAP patients (OR = 1.306, 95% CI: 1.020–1.673, *P* = 0.034) (**Table 3**). CURB-65, hypoproteinemia, heart failure, COPD, and lung cancer were also independent risk factors for respiratory failure in hospitalized CAP patients.

**Table 3:** The risk factors of respiratory failure in community acquired pneumonia inpatients.

Variable		OR	95%CI	*P*
CURB-65	Low risk group	1		
	Intermediate risk group	1.478	1.138~1.920	0.003
	High risk group	3.785	2.768~5.175	<0.001
Lung cancer		1.753	1.163~2.643	0.007
COPD		2.525	1.784~3.575	<0.001
Heart failure		2.152	1.673~2.767	<0.001
Hypoproteinemia		2.098	1.643~2.679	<0.001
Type 2 diabetes		1.306	1.020~1.673	0.034

Multiple logistic regression analysis, respiratory failure was considered as the dependent variables in a multiple logistic regression analysis with sex, age, ethnicity, CURB-65, type 2 diabetes, lung cancer, COPD, asthma, CHD, heart failure, CKD, hypoproteinemia, stroke and TIA as independent variables. OR, odds ratio; CI, confidence interval; COPD, chronic obstructive pulmonary disease; CHD, coronary heart disease; CKD, chronic kidney disease; TIA, transient ischemic attack.

A total of 130 patients died in this study, resulting in an in-hospital mortality rate of 5.3% among CAP patients. The in-hospital mortality rate was 4.3% in non-diabetic CAP patients and 8.4% in T2DM patients. The in-hospital mortality rate of T2DM patients was significantly higher than that of non-diabetic patients (*χ*^2^ = 14.353, *P* < 0.001). Multivariate logistic regression analysis demonstrated that T2DM was an independent risk factor for in-hospital death in hospitalized CAP patients (OR = 1.713, 95% CI: 1.157–2.536, *P* = 0.007) (**Table 4**). CURB-65, hypoproteinemia, heart failure, and lung cancer were also independent risk factors for in-hospital death in hospitalized CAP patients.

**Table 4:** The risk factors of death in community acquired pneumonia inpatients.

Variable		OR	95%CI	*P*
CURB-65	Low risk group	1		
	Intermediate risk group	1.847	1.142~2.987	0.012
	High risk group	4.319	2.560~7.287	<0.001
Lung cancer		5.021	2.937~8.585	<0.001
Heart failure		2.206	1.480~3.289	<0.001
Hypoproteinemia		2.756	1.881~4.040	<0.001
Type 2 diabetes		1.713	1.157~2.536	0.007

Multiple logistic regression analysis, death was considered as the dependent variables in a multiple logistic regression analysis with sex, age, ethnicity, CURB-65, type 2 diabetes, lung cancer, COPD, asthma, CHD, heart failure, CKD, hypoproteinemia, stroke and TIA as independent variables. OR, odds ratio; CI, confidence interval; COPD, chronic obstructive pulmonary disease; CHD, coronary heart disease; CKD, chronic kidney disease; TIA, transient ischemic attack.

After PSM, 559 cases from each group were well matched by a 1:1 matching algorithm. There were no statistically significant differences in the levels of age and CURB-65, and the distribution of gender and ethnicity between the two groups (*P* > 0.05). The incidences of septic shock, respiratory failure, and in-hospital mortality in T2DM patients were still significantly higher than that in non-diabetic patients (*P* < 0.05) (**Supplementary Table 1**).

Stratified analyses by gender, age and CURB-65 score were further performed to explore the association between T2DM and in-hospital mortality (**Supplementary Table 2**). In gender stratification, T2DM was significantly linked to elevated in-hospital mortality in both male (OR = 1.800, 95%CI: 1.114–2.909, *P* = 0.016) and female patients (OR = 2.437, 95%CI: 1.352–4.393, *P* = 0.003). For age subgroups, no mortality events were observed in diabetic patients aged below 50 years. A marginally increased mortality risk was found in the 50–59 years group (OR = 5.974, 95%CI: 0.975–36.597, *P* = 0.053) and the 60–69 years group (OR = 2.302, 95%CI: 0.987–5.371, *P* = 0.054), while no significant correlation was detected in patients aged 70 years and older (*P* > 0.05). Stratified by CURB-65 risk grade, T2DM was an independent risk factor for in-hospital mortality in patients with low risk group (OR = 2.150, 95%CI: 1.092–4.233, *P* = 0.027) and intermediate risk group (OR = 1.966, 95%CI: 1.066–3.626, *P* = 0.030), yet no significant difference was seen in the high-risk group (OR = 1.133, 95%CI: 0.563–2.280, *P* = 0.727).

## Discussion

The results of this study showed that patients with T2DM complicated by CAP had higher proportions of septic shock and respiratory failure. More of these patients required mechanical ventilation support and ICU admission, leading to prolonged hospital stays and increased medical costs. These findings are consistent with those of international studies. A Swiss multicenter study demonstrated that diabetes is an independent risk factor for prolonged hospital stay in CAP patients (24). Data from Portugal also indicated that diabetes prolonged the average hospital stay of CAP patients by 0.8 days (20). Additionally, data from the United States Medical Expenditure Panel Surveys showed that the average medical cost per T2DM patient with pneumonia was $11,931, which was higher than the $7,751 for non-diabetic pneumonia patients (25). In the present study, T2DM patients had an average hospital stay prolonged by 2 days and an increase in medical costs of approximately 5,000 RMB.

With the prevalence of T2DM, T2DM patients complicated by CAP have become a very common clinical issue. However, current research findings on the prognosis of T2DM patients with CAP remain inconsistent. A Danish cohort study showed that T2DM patients had higher 30-day and 90-day in-hospital mortality rates than the control group (26). A Finnish study reported that diabetic patients with hospitalized CAP had a higher 30-day mortality rate than non-diabetic patients (27). A national hospital survey in Portugal also found that diabetic CAP patients had a higher in-hospital mortality rate than non-diabetic patients (20). Recent research also showed that diabetes increased CAP incidence (relative risk 1.73), hospitalization (+30%-50%), and 30-day mortality (odds ratio 1.67) (19).[PMID: 41805689] Co-morbidity of diabetes was also independently associated with poor discharge outcomes in CAP patients (28). In contrast, a survey of elderly hospitalized CAP patients in the United States showed that diabetic patients had a higher mortality risk than non-diabetic patients (OR = 1.27), but no increase in mortality risk was observed after adjusting for relevant confounding factors (OR = 0.96) (29). A study from Xi’an, China, reported similar results (30). A retrospective observational study using Spain’s national hospital discharge database from 2004 to 2013 revealed that T2DM patients had a higher incidence of CAP than non-diabetic patients, but no increase in mortality (21). An Iranian study reported that the mortality rate for the diabetic patients was not significantly higher (16.8% vs. 15.7%) (31). The discrepancies between different studies may be attributed to factors such as the ethnicity, age composition, and comorbidities of the study populations.

The results of this study showed that T2DM patients with CAP had a higher in-hospital mortality rate than non-diabetic patients. T2DM patients were older and had a significantly higher proportion of comorbidities than non-diabetic patients. To adjust for these confounding factors, multivariate logistic regression analysis was performed. The results indicated that T2DM was independently associated with in-hospital mortality in CAP patients after adjusting for age and other relevant confounding factors, including pneumonia severity score CURB-65. This result was further confirmed by the PSM analysis after adjustment for confounding factors.

Stratified analysis was also conducted. By gender, T2DM patients with CAP had a higher in-hospital mortality rate than non-diabetic CAP patients in both male and female subgroups. By age group, in-hospital mortality of CAP increased along with age in both T2DM and non-diabetic populations. Mortality among CAP patients complicated with T2DM began to rise from the age of 50 years, an earlier turning point than that seen in non-diabetic patients. According to univariate logistic regression analysis, the OR value for in-hospital mortality between the two groups declined as age increased, indicating a gradual convergence of mortality disparities with aging. Based on the present data, we speculate that T2DM patients with CAP are likely to develop higher death risk at an earlier life stage. It is worth noting that younger age groups had relatively small sample sizes and few mortality events, which may affect the reliability of the above trend. Therefore, the mortality rate of CAP patients in these age groups requires confirmation with larger-scale epidemiological data. Stratified analysis based on CURB-65 scores revealed that T2DM was significantly associated with higher in-hospital mortality among CAP patients at low and intermediate risk group. No significant correlation was observed in patients with high risk group. These findings indicate that the adverse prognostic impact of T2DM is more prominent in patients with relatively mild pneumonia.

The severe condition and poor prognosis of T2DM patients with CAP may be related to the following mechanisms: 1. Impaired Immune Response. The immune system plays a crucial role in defending against pathogenic microbial invasion and clearance. Diabetic patients have reduced clearance of pathogenic microorganisms by airway cilia (32). Diabetes can also cause delayed recruitment and reduced adhesion of pulmonary neutrophils (33, 34). Toll-like receptors (TLRs) play an important role in the host response of patients with pulmonary infections. The expression of TLR-2 and TLR-4 in monocytes of T2DM patients is inhibited, and this inhibition is more pronounced in T2DM patients with pneumonia (35, 36). Adaptive immunity is a specific immune response to pathogenic microorganisms, with more efficient effects. Hyperglycemia in T2DM patients leads to decreased antibody titers against pneumococcal surface protein A and reduced opsonin activity, and T2DM is associated with impaired antibody responses (37). T2DM patients have weakened pathogen-specific memory CD4+ cell and Th17 cell responses, and reduced memory T cells in response to Streptococcus pneumoniae stimulation, which is closely related to blood glucose control (38). In addition, long-term chronic metabolic disorders in diabetic patients increase the magnitude of the immune response, accelerating immune system aging and leading to premature immune dysfunction (39). This premature immune dysfunction may be related to the earlier onset of increased in-hospital mortality risk in T2DM patients with CAP. 2. Altered Airway Glucose Homeostasis. Hyperglycemia increases the glucose concentration in airway surface fluid by enhancing the glucose concentration gradient across the airway epithelium. At the same time, hyperglycemia can damage the tight junctions between airway epithelial cells, leading to increased glucose leakage into the airways. *In vitro* experiments, animal studies, and human studies have all indicated that elevated glucose levels in airway surface fluid promote the growth of Staphylococcus aureus, Pseudomonas aeruginosa, and other Gram-negative bacteria. In humans, airway glucose homeostasis is closely associated with clinical respiratory infectious diseases (40). 3. Decreased Lung Function. T2DM can cause decreased lung function, manifested as reduced pulmonary ventilation and diffusion function. A meta-analysis showed that diabetes leads to decreased forced vital capacity (FVC), forced expiratory volume in the first second (FEV1), and carbon monoxide diffusion function (41). An increasing number of prospective cohort studies have confirmed that FVC decreases more significantly in diabetic patients with prolonged disease duration (42, 43). The results of our study also showed that T2DM patients with CAP had a higher proportion of respiratory failure and more required mechanical ventilation support. A Canadian study also found that respiratory failure is the most common complication in diabetic patients with CAP (44). However, these potential mechanisms still need to be further investigated and verified.

This study has the following limitations. Firstly, an important limitation is the lack of microbiological and treatment-related data. We failed to obtain information on CAP causative pathogens, while the microbial spectrum may differ between diabetic patients and non-diabetic individuals (45, 46). Meanwhile, key therapeutic variables including antimicrobial treatment, timing of therapy initiation, corticosteroid use and adequacy of antimicrobial therapy were not assessed. These unrecorded variables act as potential confounders and may exert impacts on patient clinical outcomes. Secondly, future investigations should explore potential risk factors, including glycemic status, duration of diabetes, diabetes complications, and antidiabetic treatment, that may affect the mortality inT2DM patients with CAP.

In conclusion, CAP patients with T2DM have higher proportions of septic shock and respiratory failure, and more require mechanical ventilation support and ICU admission. T2DM patients have longer hospital stays and higher medical costs. T2DM is closely associated with increased in-hospital mortality in CAP patients, especially for patients with low to intermediate risk. Given these characteristics of T2DM patients with CAP, establishing effective assessment methods, identifying high-risk patients early, and strengthening management to reduce the mortality rate of T2DM patients with CAP are of great clinical significance.

## Data Availability

The raw data supporting the conclusions of this article will be made available by the authors, without undue reservation.

## References

[1] Jain S Self WH Wunderink RG Fakhran S Balk R Bramley AM . Community-acquired pneumonia requiring hospitalization among U.S. adults. N Engl J Med. (2015) 373:415–27. doi: 10.1056/nejmoa1500245 26172429 PMC4728150

[2] Partouche H Lepoutre A Vaure CBD Poisson T Toubiana L Gilberg S . Incidence of all-cause adult community-acquired pneumonia in primary care settings in France. Med Mal Infect. (2018) 48:389–95. doi: 10.1016/j.medmal.2018.02.012 29656842

[3] Lopardo GD Fridman D Raimondo E Albornoz H Lopardo A Bagnulo H . Incidence rate of community-acquired pneumonia in adults: a population-based prospective active surveillance study in three cities in South America. BMJ Open. (2018) 8:e019439. doi: 10.1136/bmjopen-2017-019439 29643153 PMC5898349

[4] Heo JY Seo YB Choi WS Lee J Yoon JG Lee SN . Incidence and case fatality rates of community-acquired pneumonia and pneumococcal diseases among Korean adults: Catchment population-based analysis. PLoS One. (2018) 13:e0194598. doi: 10.1371/journal.pone.0194598 29596444 PMC5875769

[5] Kosar F Alici DE Hacibedel B Arpınar Yigitbas B Golabi P Cuhadaroglu C . Burden of community-acquired pneumonia in adults over 18 y of age. Hum Vaccin Immunother. (2017) 13:1673–80. doi: 10.1080/21645515.2017.1300730 28281915 PMC5512757

[6] Corrado RE Lee D Lucero DE Varma JK Vora NM . Burden of adult community-acquired, health-care-associated, hospital-acquired, and ventilator-associated pneumonia: New York City, 2010 to 2014. Chest. (2017) 152:930–42. doi: 10.1016/j.chest.2017.04.162 28455128

[7] Divino V Schranz J Early M Shah H Jiang M DeKoven M . The annual economic burden among patients hospitalized for community-acquired pneumonia (CAP): a retrospective US cohort study. Curr Med Res Opin. (2020) 36:151–60. doi: 10.1080/03007995.2019.1675149 31566005

[8] McLaughlin JM Johnson MH Kagan SA Baer SL . Clinical and economic burden of community-acquired pneumonia in the Veterans Health Administration, 2011: a retrospective cohort study. Infection. (2015) 43:671–80. doi: 10.1007/s15010-015-0789-3 25980561 PMC4656694

[9] Choi MJ Song JY Noh JY Yoon JG Lee SN Heo JY . Disease burden of hospitalized community-acquired pneumonia in South Korea: Analysis based on age and underlying medical conditions. Med (Baltimore). (2017) 96:e8429. doi: 10.1097/md.0000000000008429 29095281 PMC5682800

[10] Chinese Medical Association . Guideline for primary care of adult community acquired pneumonia (2018). Chin J Gen Pract. (2018) 18:117–26. doi: 10.3760/cma.j.issn.1671-7368.2019.02.005

[11] Han X Zhou F Li H Xing X Chen L Wang Y . Effects of age, comorbidity and adherence to current antimicrobial guidelines on mortality in hospitalized elderly patients with community-acquired pneumonia. BMC Infect Dis. (2018) 18:192. doi: 10.1186/s12879-018-3098-5 29699493 PMC5922029

[12] Espinoza R Silva J Bergmann A de Oliveira Melo U Calil FE Santos RC . Factors associated with mortality in severe community-acquired pneumonia: A multicenter cohort study. J Crit Care. (2019) 50:82–6. doi: 10.1016/j.jcrc.2018.11.024 30502687

[13] File TM Jr. Marrie TJ . Burden of community-acquired pneumonia in North American adults. Postgrad Med. (2010) 122:130–41. doi: 10.3810/pgm.2010.03.2130 20203464

[14] Ito A Ishida T Tokumasu H Washio Y Yamazaki A Ito Y . Prognostic factors in hospitalized community-acquired pneumonia: a retrospective study of a prospective observational cohort. BMC Pulm Med. (2017) 17:78. doi: 10.1186/s12890-017-0424-4 28464807 PMC5414343

[15] International Diabetes Federation . IDF Diabetes Atlas Teb, Belgium (2019). Available online at: http://www.diabetesatlas.org (Accessed January 23, 2020).

[16] Sandler M . Is the lung a 'target organ' in diabetes mellitus? Arch Intern Med. (1990) 150:1385–8. 2196023

[17] Koziel H Koziel MJ . Pulmonary complications of diabetes mellitus. Pneumonia. Infect Dis Clin North Am. (1995) 9:65–96. 7769221

[18] Torres A Blasi F Dartois N Akova M . Which individuals are at increased risk of pneumococcal disease and why? Impact of COPD, asthma, smoking, diabetes, and/or chronic heart disease on community-acquired pneumonia and invasive pneumococcal disease. Thorax. (2015) 70:984–9. doi: 10.1136/thoraxjnl-2015-206780 26219979 PMC4602259

[19] Xie Y Zhang A Wang Y Wang R . Community-acquired pneumonia in patients with diabetes: Narrative review. JMIR Diabetes. (2026) 11:e82215. doi: 10.2196/82215 41805689 PMC12975005

[20] Martins M Boavida JM Raposo JF Froes F Nunes B Ribeiro RT . Diabetes hinders community-acquired pneumonia outcomes in hospitalized patients. BMJ Open Diabetes Res Care. (2016) 4:e000181. doi: 10.1136/bmjdrc-2015-000181 27252873 PMC4879333

[21] López-de-Andrés A de Miguel-Díez J Jiménez-Trujillo I Hernández-Barrera V de Miguel-Yanes JM Méndez-Bailón M . Hospitalisation with community-acquired pneumonia among patients with type 2 diabetes: an observational population-based study in Spain from 2004 to 2013. BMJ Open. (2017) 7:e013097. doi: 10.1136/bmjopen-2016-013097 28057653 PMC5223662

[22] Cao B Huang Y She DY Cheng QJ Fan H Tian XL . Diagnosis and treatment of community-acquired pneumonia in adults: 2016 clinical practice guidelines by the Chinese Thoracic Society, Chinese Medical Association. Clin Respir J. (2018) 12:1320–60. doi: 10.1111/crj.12674 28756639 PMC7162259

[23] Lim WS van der Eerden MM Laing R Boersma WG Karalus N Town GI . Defining community acquired pneumonia severity on presentation to hospital: an international derivation and validation study. Thorax. (2003) 58:377–82. doi: 10.1136/thorax.58.5.377 12728155 PMC1746657

[24] Suter-Widmer I Christ-Crain M Zimmerli W Albrich W Mueller B Schuetz P . Predictors for length of hospital stay in patients with community-acquired pneumonia: results from a Swiss multicenter study. BMC Pulm Med. (2012) 12:21. doi: 10.1186/1471-2466-12-21 22607483 PMC3475050

[25] Liu K Lee GC . Healthcare utilisation and cost expenditures for pneumonia in individuals with diabetes mellitus in the USA. Epidemiol Infect. (2019) 147:e212. doi: 10.1017/s0950268819000979 31364575 PMC6624864

[26] Kornum JB Thomsen RW Riis A Lervang HH Schønheyder HC Sørensen HT . Type 2 diabetes and pneumonia outcomes: a population-based cohort study. Diabetes Care. (2007) 30:2251–7. doi: 10.2337/dc06-2417 17595354

[27] Koskela HO Salonen PH Romppanen J Niskanen L . Long-term mortality after community-acquired pneumonia--impacts of diabetes and newly discovered hyperglycaemia: a prospective, observational cohort study. BMJ Open. (2014) 4:e005715. doi: 10.1136/bmjopen-2014-005715 25146717 PMC4156798

[28] Chen S Hou C Kang Y Li D Rong J Li Z . Factors affecting hospital discharge outcomes in patients with community-acquired pneumonia: A retrospective epidemiological study (2014-2021). Am J Med Sci. (2023) 366:143–9. doi: 10.1016/j.amjms.2023.05.008 37220846

[29] Kaplan V Angus DC Griffin MF Clermont G Scott Watson R Linde-Zwirble WT . Hospitalized community-acquired pneumonia in the elderly: age- and sex-related patterns of care and outcome in the United States. Am J Respir Crit Care Med. (2002) 165:766–72. doi: 10.1164/ajrccm.165.6.2103038 11897642

[30] Li S Li L Wang S Wu H . Clinical characteristics and risk factors of hospital mortality in elderly patients with community-acquired pneumonia. Front Med (Lausanne). (2025) 12:1512288. doi: 10.3389/fmed.2025.1512288 40177286 PMC11961441

[31] Hashemi SH Saki F Borzouei S Bawand R Soltanian A . Comprehensive comparison of clinicoradiological, laboratory, and prognostic factors of community-acquired pneumonia in diabetic and nondiabetic hospitalized patients. Turk J Med Sci. (2023) 53:1776–85. doi: 10.55730/1300-0144.5747 38813518 PMC10760569

[32] Proença de Oliveira-Maul J Barbosa de Carvalho H Goto DM Maia RM Fló C Barnabé V . Aging, diabetes, and hypertension are associated with decreased nasal mucociliary clearance. Chest. (2013) 143:1091–7. doi: 10.1378/chest.12-1183 23100111

[33] Amano H Yamamoto H Senba M Oishi K Suzuki S Fukushima K . Impairment of endotoxin-induced macrophage inflammatory protein 2 gene expression in alveolar macrophages in streptozotocin-induced diabetes in mice. Infect Immun. (2000) 68:2925–9. doi: 10.1128/iai.68.5.2925-2929.2000 10768990 PMC97505

[34] Andreasen AS Pedersen-Skovsgaard T Berg RM Svendsen KD Feldt-Rasmussen B Pedersen BK . Type 2 diabetes mellitus is associated with impaired cytokine response and adhesion molecule expression in human endotoxemia. Intensive Care Med. (2010) 36:1548–55. doi: 10.1007/s00134-010-1845-1 20229041

[35] Que Y Shen X . Changes in blood monocyte Toll-like receptor and serum surfactant protein A reveal a pathophysiological mechanism for community-acquired pneumonia in patients with type 2 diabetes. Intern Med J. (2016) 46:213–9. doi: 10.1111/imj.12978 26648341

[36] Khondkaryan L Margaryan S Poghosyan D Manukyan G . Impaired inflammatory response to LPS in type 2 diabetes mellitus. Int J Inflam. (2018) 2018:2157434. doi: 10.1155/2018/2157434 29568481 PMC5820544

[37] Mathews CE Brown EL Martinez PJ Bagaria U Nahm MH Burton RL . Impaired function of antibodies to pneumococcal surface protein A but not to capsular polysaccharide in Mexican American adults with type 2 diabetes mellitus. Clin Vaccine Immunol. (2012) 19:1360–9. doi: 10.1128/cvi.00268-12 22761295 PMC3428395

[38] Martinez PJ Mathews C Actor JK Hwang SA Brown EL De Santiago HK . Impaired CD4+ and T-helper 17 cell memory response to Streptococcus pneumoniae is associated with elevated glucose and percent glycated hemoglobin A1c in Mexican Americans with type 2 diabetes mellitus. Transl Res. (2014) 163:53–63. doi: 10.1016/j.trsl.2013.07.005 23927943 PMC3954646

[39] Moura J Madureira P Leal EC Fonseca AC Carvalho E . Immune aging in diabetes and its implications in wound healing. Clin Immunol. (2019) 200:43–54. doi: 10.1016/j.clim.2019.02.002 30735729 PMC7322932

[40] Baker EH Baines DL . Airway glucose homeostasis: A new target in the prevention and treatment of pulmonary infection. Chest. (2018) 153:507–14. doi: 10.1016/j.chest.2017.05.031 28610911

[41] van den Borst B Gosker HR Zeegers MP Schols AM . Pulmonary function in diabetes: a metaanalysis. Chest. (2010) 138:393–406. doi: 10.1378/chest.09-2622 20348195

[42] Yeh HC Punjabi NM Wang NY Pankow JS Duncan BB Cox CE . Cross-sectional and prospective study of lung function in adults with type 2 diabetes: the Atherosclerosis Risk in Communities (ARIC) study. Diabetes Care. (2008) 31:741–6. doi: 10.2337/dc07-1464 18056886 PMC2773203

[43] Sonoda N Morimoto A Tatsumi Y Asayama K Ohkubo T Izawa S . A prospective study of the impact of diabetes mellitus on restrictive and obstructive lung function impairment: The Saku study. Metabolism. (2018) 82:58–64. doi: 10.1016/j.metabol.2017.12.006 29288691

[44] Bader MS Yi Y Abouchehade K Haroon B Bishop LD Hawboldt J . Community-acquired pneumonia in patients with diabetes mellitus: Predictors of complications and length of hospital stay. Am J Med Sci. (2016) 352:30–5. doi: 10.1016/j.amjms.2016.02.032 27432032

[45] Liu BY Zhang D Fan Z Jin JJ Li CH Guo RN . Role of clinical features, pathogenic and etiological characteristics of community-acquired pneumonia with type 2 diabetes mellitus in early diagnosis. Endocr Metab Immune Disord Drug Targets. (2024) 24:958–66. doi: 10.2174/0118715303273741231117060753 38686909

[46] Liu X Zhao Q He X Min J Yao RSY Chen Z . Clinical characteristics and microbial signatures in the lower airways of diabetic and nondiabetic patients with pneumonia. J Thorac Dis. (2024) 16:5262–73. doi: 10.21037/jtd-24-490 39268134 PMC11388247

