# Supplementary material for: The effect of type 2 diabetes on the prognosis of community-acquired pneumonia
Source: Front Endocrinol (Lausanne). 2026 Jul 14;17:1878364. doi: 10.3389/fendo.2026.1878364 (PMC13407086; doi:10.3389/fendo.2026.1878364)
Supplement: Supplementary file 1 [file Table1.doc]

Supplementary Table 1 The characteristics of community acquired pneumonia inpatients with and without type 2 diabetes (propensity score matching).

| Variables |  | Non-diabetes group  (N=559) | Type 2 diabetes group  (N=559) | *t* or *χ*^2^ | *P* |
| --- | --- | --- | --- | --- | --- |
| Sex(males/females) |  | 338/221 | 320/239 | 1.197 | 0.274 |
| Age (years) |  | 71.9±12.3 | 71.7±11.8 | 0.285 | 0.776 |
| Ethnicity[n(%)] | Han | 549(98.2) | 536(95.9) | 5.277 | 0.071 |
|  | Other | 7(1.3) | 16(2.9) |  |  |
|  | Unknown | 3(0.5) | 7(1.2) |  |  |
| CURB-65 Score |  | 1.0(1.0～2.0) | 1.0(1.0～2.0) | 1.448 | 0.148 |
| CURB-65 Grade[n(%)] | Low risk group | 332(59.4) | 313(56.0) | 3.737 | 0.154 |
|  | Intermediate risk group | 174(31.1) | 173(30.9) |  |  |
|  | High risk group | 53(9.5) | 73(13.1) |  |  |
| Hospital length of stay[days] |  | 9.0(7.0～14.0) | 11.0(8.0～16.0) | 5.221 | <0.001 |
| Hospital cost[CNY] |  | 12360(7834～20315) | 16170(10423～28679) | 6.512 | <0.001 |
| Mechanical  ventilation[n(%)] |  | 50(8.9) | 73(13.1) | 4.832 | 0.028 |
| ICU admissions[n(%)] |  | 68(12.2) | 97(17.4) | 5.979 | 0.014 |
| Septic shock[n(%)] |  | 21(3.8) | 36(6.4) | 4.159 | 0.041 |
| Respiratory failure[n(%)] |  | 96(17.2) | 127(22.7) | 5.383 | 0.020 |
| Death[n(%)] |  | 23(4.1) | 47(8.4) | 8.778 | 0.003 |

Propensity score matching was also used to minimize the effect of confounding factors. A one-to-one nearest neighbor matching algorithm was applied using a caliper width of 0.05. The following variables were selected to generate the propensity score: age, gender, ethnicity, and CURB-65. Finally, 559 matched pairs were generated and applied to further analyses. Numerical data were expressed as mean±SD, and when not normally distributed, they were expressed as medians (IQR) and Mann-Whitney U test was used for comparing measurement data between groups. COPD: chronic obstructive pulmonary disease; CHD: coronary heart disease; TIA: transient ischemic attack; CKD: chronic kidney disease; CNY: China Yuan; ICU: intensive care unit; SD: standard deviation; IQR: interquartile range.

Supplementary Table 2 Association between type 2 diabetes and in-hospital mortality in community acquired pneumonia inpatients (stratified analysis).

| Stratified by |  | In-hospital mortality [n(%)] | OR | 95%CI | *P* |
| --- | --- | --- | --- | --- | --- |
| Gender |  |  |  |  |  |
| Males (N=1429) | Non-diabetes group  (N=1109) | 54(4.9) | 1 |  |  |
|  | Type 2 diabetes group  (N=320) | 27(8.4) | 1.800 | 1.114～2.909 | 0.016 |
| Females (N=1042) | Non-diabetes group  (N=803) | 29(3.6) | 1 |  |  |
|  | Type 2 diabetes group  (N=239) | 20(8.4) | 2.437 | 1.352～4.393 | 0.003 |
| Age |  |  |  |  |  |
| ～39 years (N=263) | Non-diabetes group  (N=257) | 3(1.2) | 1 |  |  |
|  | Type 2 diabetes group  (N=6) | 0(0.0) | NA |  |  |
| 40～49 years (N=160) | Non-diabetes group  (N=144) | 1(0.7) | 1 |  |  |
|  | Type 2 diabetes group  (N=16) | 0(0.0) | NA |  |  |
| 50～59 years (N=289) | Non-diabetes group  (N=229) | 2(0.9) | 1 |  |  |
|  | Type 2 diabetes group  (N=60) | 3(5.0) | 5.974 | 0.975～36.597 | 0.053 |
| 60～69 years (N=558) | Non-diabetes group  (N=414) | 13(3.1) | 1 |  |  |
|  | Type 2 diabetes group  (N=144) | 10(6.9) | 2.302 | 0.987～5.371 | 0.054 |
| 70～79 years (N=563) | Non-diabetes group  (N=387) | 23(5.9) | 1 |  |  |
|  | Type 2 diabetes group  (N=176) | 15(8.5) | 1.474 | 0.750～2.900 | 0.261 |
| 80 years～(N=638) | Non-diabetes group  (N=481) | 41(8.5) | 1 |  |  |
|  | Type 2 diabetes group  (N=157) | 19(12.1) | 1.478 | 0.830～2.630 | 0.184 |
| CURB-65 |  |  |  |  |  |
| Low risk group (N=1629) | Non-diabetes group  (N=1316) | 26(2.0) | 1 |  |  |
|  | Type 2 diabetes group  (N=313) | 13(4.2) | 2.150 | 1.092～4.233 | 0.027 |
| Intermediate risk group (N=590) | Non-diabetes group  (N=417) | 26(6.2) | 1 |  |  |
|  | Type 2 diabetes group  (N=173) | 20(11.6) | 1.966 | 1.066～3.626 | 0.030 |
| High risk group (N=252) | Non-diabetes group  (N=179) | 31(17.3) | 1 |  |  |
|  | Type 2 diabetes group  (N=73) | 14(19.2) | 1.133 | 0.563～2.280 | 0.727 |

Univariate logistic regression analysis, in-hospital mortality was considered as the dependent variable. OR: odds ratio; CI: confidence interval.
